# Original delayed-start ovarian stimulation protocol with a gonadotropin-releasing hormone antagonist, medroxyprogesterone acetate, and high-dose gonadotropin for poor responders and patients with poor-quality embryos

**DOI:** 10.3389/fendo.2023.1277873

**Published:** 2023-10-31

**Authors:** Kazuhiro Takeuchi, Yuji Orita, Tokiko Iwakawa, Yukari Kuwatsuru, Yuko Kuroki, Yumiko Fukumoto, Yamato Mizobe, Mari Tokudome, Harue Moewaki

**Affiliations:** Takeuchi Ladies Clinic/Center for Reproductive Medicine, Kagoshima, Japan

**Keywords:** delayed start, gonadotropin-releasing hormone antagonist, medroxyprogesterone acetate, poor responder, poor-quality embryo

## Abstract

**Introduction:**

The delayed-start gonadotropin-releasing hormone antagonist protocol seems effective for patients who are poor ovarian responders, but there are insufficient data on whether it is also effective for patients with poor-quality embryos and low rates of good blastocyst formation. Specifically, the effectiveness of delayed-start gonadotropin-releasing hormone antagonists with progesterone has not been adequately investigated. Therefore, we compared the efficacy of the original delayed-start gonadotropin-releasing hormone antagonist protocol using medroxyprogesterone acetate (MPA) and high-dose gonadotropin in patients with poor ovarian response.

**Methods:**

Overall, 156 patients with recurrent assisted reproductive technology failure who underwent the original protocol were included. They received cetrorelix acetate (3 mg) and MPA (10 mg) on cycle day 3, and high-dose gonadotropin was initiated on day 11. When the leading follicle reached 14 mm, ganirelix acetate (0.25 mg) was administered until the trigger day. The number of oocytes retrieved, metaphase II (MII) oocytes, two pronuclear (2PN) zygotes, and good blastocysts and live birth rates were compared between the previous (Cycle A) and original (Cycle B) cycles in three groups (Group A, all patients; Group B, poor responders; and Group C, patients with poor-quality embryos).

**Results:**

In Group A (n=156), the number of MII oocytes (3.6 ± 3.3 versus 4.5 ± 3.6), 2PN zygotes (2.8 ± 2.9 versus 3.8 ± 3.1), good blastocysts (0.5 ± 0.9 versus 1.2 ± 1.6), and live birth rates (0.6 versus 24.4) significantly increased in Cycle B. Similar results were obtained in Group B (n=83; 2PN zygotes [1.7 ± 1.7 versus 2.3 ± 1.8], good blastocysts [0.4 ± 0.7 versus 0.9 ± 1.3], live birth rates [0 versus 18.1]) and Group C (n=73; MII oocytes [5.1 ± 3.8 versus 6.6 ± 4.0], 2PN zygotes [4.0 ± 3.4 versus 5.4 ± 3.4], good blastocysts [0.7 ± 1.1 versus 1.6 ± 1.9], and live birth rates [1.4 versus 31.5]).

**Conclusion:**

This original protocol increased the number of MII oocytes retrieved, 2PN zygotes, good blastocysts, and live birth rates in both poor responders and in patients with poor-quality embryos.

## 1 Introduction

The main goal of controlled ovarian stimulation (COS) in assisted reproductive technology (ART), including conventional *in vitro* fertilization and intracytoplasmic sperm injection cycles, is to achieve live births in the least number of cycles. For this purpose, many COS protocols have been established to retrieve multiple mature oocytes, increase fertilized embryo count, and obtain good blastocysts according to the patients’ backgrounds (1), particularly in patients with poor ovarian response (5.6–35.1% of ART patients) (2). Low COS response is a crucial concern in poor responders and patients with poor-quality embryos. To overcome this problem, several COS protocols have been reported, including delayed-start gonadotropin-releasing hormone (GnRH) antagonists (3–13), microdose GnRH agonists (14–16), and long GnRH agonists (17, 18). Among these, a current meta-analysis recommended a delayed-start GnRH antagonist protocol (13) for poor responders. In addition to the improvement in the number of mature oocytes, the clinical pregnancy rate was increased significantly (relative risk: 2.90, [95% confidence interval: 1.52–5.51], *P* = 0.001) (11). The delayed-start GnRH antagonist protocol seems effective for poor responders (3, 4, 7–13), but there are insufficient data on whether it is also effective for patients with poor-quality embryos and low rates of good blastocyst formation. The delayed-start GnRH antagonist protocol has some variations, such as the combination with estrogen or progesterone and the frequency of GnRH antagonist administration as a pretreatment. Frankfurter et al. reported the effectiveness of a delayed-start GnRH antagonist with progesterone (3). Some reports have investigated delayed-start GnRH antagonists with estrogen (7–10), but there are no additional reports investigating the effectiveness of delayed-start GnRH antagonists with progesterone. In this study, an original protocol, modified from Frankfurter’s protocol, was evaluated. This study aimed to investigate the efficiency of this original delayed-start GnRH antagonist with progesterone in patients with poor ovarian response, including poor responders according to the Bologna criteria (19) and patients with poor-quality embryos.

## 2 Methods

### 2.1 Study design and population

**Figure 1:**
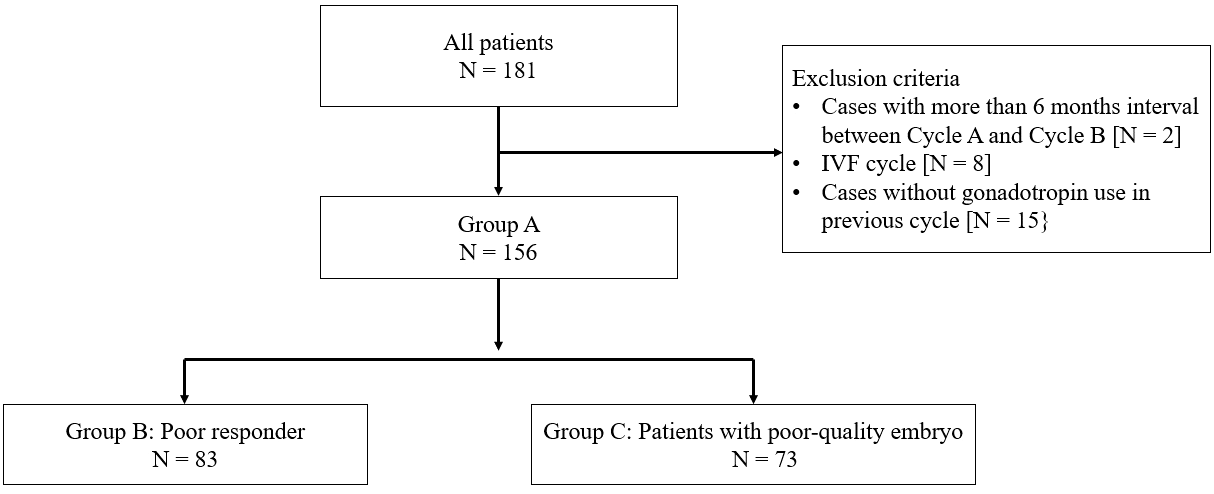
Patient selection flow chart. IVF, *in vitro* fertilization.

Patients who underwent the original delayed-start GnRH antagonist with the progesterone protocol whose previous COS cycle resulted in poor outcomes (low rate of metaphase II [MII] oocytes, fertilization) from May 2015 to January 2021 were included. Cases with an interval >6 months between Cycle A and Cycle B, *in vitro* fertilization cycles, and without gonadotropin usage in Cycle A were excluded. Finally, 156 patients who underwent intracytoplasmic sperm injection were included in this study (**Figure 1**). To investigate protocol effectiveness, the previous COS cycle (Cycle A) and the delayed-start GnRH antagonist with progesterone cycle (Cycle B) were compared. In addition, patients were divided into three groups, and the outcomes were compared in each group: Group A, all patients; Group B, poor responders according to the Bologna criteria (19) and Group C, patients with poor-quality embryos who did not satisfy the Bologna criteria (**Figure 1**).

This study was approved by the institutional review board of Takeuchi Ladies Clinic/Center for Reproductive Medicine (Number: 23-201) and was conducted in accordance with the 2013 Declaration of Helsinki. We announced this study in displays and on the hospital’s homepage and provided an opt-out option for patients.

### 2.2 Data collection

All data were collected from the patients’ medical records. The baseline characteristics of all patients, including maternal age, number of previous oocyte pick-up (OPU) cycles and previous embryo transfer (ET) cycles including previous clinics, and anti-Müllerian hormone levels in Cycle B, were collected. The period from day 1 of the menstrual cycle to the OPU day; dose of gonadotropin, estradiol (E2), and progesterone (P4) on the trigger day; diameter of the leading follicle on the trigger day; number of retrieved oocytes; number of MII oocytes; rate of MII oocytes; number of two pronuclear (2PN) zygotes; number of morphologically good blastocysts; implantation rate; clinical pregnancy (defined as detection of the gestational sac) rate; and live birth rate were investigated. The reasons for cancellation were also investigated and divided into the following stages: OPU=0, MII=0, and 2PN=0.

### 2.3 Outcome measures

The primary outcome was the efficacy of the protocol which was assessed by comparing the period from day 1 of the menstrual cycle to the OPU day; dose of gonadotropin, E2, and P4 on the trigger day; diameter of the leading follicle on the trigger day; number of retrieved oocytes; number of MII oocytes; rate of MII oocytes; number of 2PN zygotes; number of morphologically good blastocysts (defined as better than 3BB according to the Gardner blastocyst grading system); implantation rate; clinical pregnancy (defined as detection of the gestational sac) rate; and live birth rate between Cycle A and Cycle B in all patients (Group A). The rates of OPU=0, MII=0, and 2PN=0 were also compared. These parameters were also compared between Groups B and C to investigate their efficiency in poor responders and patients with poor-quality embryos.

### 2.4 Ovarian stimulation protocols

Cycle A: There were 87 patients with the antagonist protocol, 68 with the agonist protocol (4 patients with the long protocol and 64 patients with the short/ultrashort protocol), and 1 with progestin-primed ovarian stimulation. When the leading follicle reached 18–20 mm in diameter and more than two follicles reached 18 mm in diameter, the trigger was administered, considering the patient’s background and the outcome of the past COS cycle, as appropriate (3000–10,000 IU of human chorionic gonadotropin, a GnRH agonist, and a dual trigger). Transvaginal OPU was performed 34–38 h later, as appropriate, considering the patient’s outcome in the past cycle.

**Figure 2:**
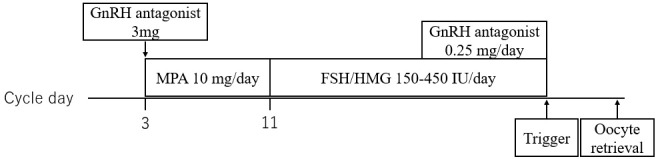
Outline of delayed-start ovarian stimulation protocol with a gonadotropin-releasing hormone antagonist, medroxyprogesterone acetate, and high-dose gonadotropin. GnRH, gonadotropin-releasing hormone; MPA, medroxyprogesterone acetate; FSH, follicle-stimulating hormone; HMG, human menopausal gonadotrophin.

Cycle B: On day 3, cetrorelix acetate (3 mg) was administered subcutaneously. From days 3–10, daily oral medroxyprogesterone acetate (MPA, 5 mg twice daily) was continued. On day 11, after ovarian suppression (E2 < 50 mg/mL) was identified, daily high-dose gonadotropin (human menopausal gonadotrophin 225–450 IU, subcutaneously) was initiated. When the leading follicle measured 14 mm, ganirelix acetate (0.25 mg, subcutaneously) was initiated and continued until the trigger day. When the leading follicle reached 18–20 mm in diameter and more than two follicles reached 18 mm in diameter, the trigger was administered considering the patient’s background and the outcome of the past COS cycle, as appropriate (3000–10,000 IU of human chorionic gonadotropin, a GnRH agonist, and a dual trigger). Transvaginal OPU was performed 34–38 h after, as appropriate, considering the patient’s outcome in the past cycle (**Figure 2**).

All oocytes underwent intracytoplasmic sperm injection, and all embryos were frozen. The embryos were then thawed and transferred into the hormone replacement cycle on days 3 or 5, as appropriate, under transabdominal ultrasound guidance.

### 2.5 Embryo transfer protocol

All transferred embryos were frozen-thawed embryo transfers during the hormone replacement cycle. Cleavage-stage embryos were frozen on day 3, and blastocysts were frozen on day 5 or 6 as appropriate. Cleavage-stage embryos were transferred 3 days after progesterone administration, and blastocysts were transferred 5 days after progesterone administration.

### 2.6 Statistical analysis

Intergroup comparisons were performed using Student’s t-test and a nonparametric test for continuous variables or Fisher’s exact test for nominal variables. Statistical significance was set at *P* < 0.05. All statistical analyses were performed using EZR (Saitama Medical Center, Jichi Medical University; http://www.jichi.ac.jp/saitama-sct/SaitamaHP.files/statmedEN.html ; Kanda, 2012) (20), which is a graphical user interface for R (The R Foundation for Statistical Computing, Vienna, Austria). More precisely, it is a modified version of R Commander designed to add statistical functions frequently used in biostatistics.

## 3 Results

Between May 2015 and January 2021, 181 patients received a delayed GnRH antagonist according to the progesterone protocol. Eleven patients were excluded based on the exclusion criteria described above, and 156 patients were included in this study. There were 83 poor responders according to the Bologna criteria (Group B) and 73 patients with poor-quality embryos who did not meet the Bologna criteria (Group C). No surgically obtained sperms or donated sperms were used in the protocols. In Group A, the patients’ median age was 40 years [interquartile range (IQR): 37–42], the median anti-Mullerian hormone level was 1.04 ng/mL [IQR: 0.48–1.83], the median number of previous OPU cycles was three [IQR: 3–5], and the median number of previous ET cycles was one [IQR: 0–3). In Group B, the patients’ median age was 41 years [IQR: 38–42], the median anti-Mullerian hormone level was 0.53 ng/mL [IQR: 0.32–0.85], the median number of previous OPU cycles was three [IQR: 2–5], and the median number of previous ET cycles was one [IQR: 0–2.5]. In Group C, the patients’ median age was 39 years [IQR: 35–41], the median anti-Mullerian hormone level was 1.86 ng/mL [IQR: 1.30–3.54], the median number of previous OPU cycles was three [IQR: 3–4.3], and the median number of previous ET cycles was one [IQR: 0–3] (**Table 1**). The rates of cleavage-stage (day 3) transfer were not different in cycles A and B (20.4% in Cycle A versus 17.9% in Cycle B, *P* = 0.264).

**Table 1:** Background of patients.

	Group A All	Group B Poor responders	Group C Poor-quality embryos
Number of patients	156	83	73
Age, years [IQR]	40 [37-42]	41 [38-42]	39 [35-41]
AMH, ng/mL [IQR]	1.04 [0.48-1.83]	0.53 [0.32-0.85]	1.86 [1.30-3.54]
Previous OPU cycles [IQR]	3 [3-5]	3 [2-5]	3 [3-4.3]
Previous ET cycles [IQR]	1 [0-3]	1 [0-2.5]	1 [0-3]

AMH, Anti-Müllerian hormone; ET, embryo transfer; IQR, interquartile range; OPU, oocyte pick-up.

In Group A, although the number of oocytes retrieved did not increase significantly between Cycles A and B (5.5 ± 5.3 versus 6.5 ± 6.3, *P* = 0.120), the number of MII oocytes (3.6 ± 3.3 versus 4.5 ± 3.6, *P* = 0.015), number of 2PN zygotes (2.8 ± 2.9 versus 3.8 ± 3.1, *P* < 0.01), and number of good blastocysts (0.5 ± 0.9 versus 1.2 ± 1.6, *P <* 0.001) increased significantly in Cycle B. Subsequent ET rates (41.0% versus 62.8%, *P <* 0.001), implantation rates/OPU cycles (11.5% versus 39.1%, *P <* 0.0001), implantation rates/the number of embryos transferred (18.4% versus 37.2%, *P <* 0.01), clinical pregnancy rates (4.5% versus 32.1%, *P <* 0.0001), and live birth rates (0.6% versus 24.4%, *P <* 0.0001) increased significantly in Cycle B. The periods up to trigger (12.8 ± 1.8 days versus 21.2 ± 2.3 days, *P <* 0.001) and gonadotropin dose (1653 ± 678 IU versus 2088 ± 819 IU, *P <* 0.001) increased significantly in Cycle B. The mean leading follicle size (21.9 ± 4.1 mm versus 21.8 ± 2.8 mm, *P* = 0.764) and E2 level (1649 ± 1231 pg/mL versus 1633 ± 1209 pg/mL, *P* = 0.909) were not different between Cycles A and B, but the P4 level was significantly lower in Cycle B (0.68 ± 0.60 ng/mL versus 0.40 ± 0.25 ng/mL, *P <* 0.001). The cancellation rates because of OPU=0 (2.6 versus 2.6, p=1) and MII=0 (5.9 versus 2.6, p=0.256) were not different, likely because the 2PN=0 was significantly lower (9.8 versus 1.4, *P <* 0.01) in Cycle B (**Table 2**).

**Table 2:** Comparison of parameters and outcomes measured in all patients (Group A).

	Cycle A	Cycle B	P
Period up to trigger, days	12.8 ± 1.8	21.2 ± 2.3	<0.001
Duration of gonadotropin injection, days	9.6 ± 1.5	10.1 ± 2.2	0.128
Gonadotropin, IU	1653 ± 678	2088 ± 819	<0.001
Leading follicle, mm	21.9 ± 4.1	21.8 ± 2.8	0.764
Estradiol, pg/mL	1649 ± 1231	1633 ± 1209	0.909
Progesterone, ng/mL	0.68 ± 0.60	0.40 ± 0.25	<0.001
Number of oocytes	5.5 ± 5.3	6.5 ± 6.3	0.120
Number of MII oocytes	3.6 ± 3.3	4.5 ± 3.6	0.015
Number of 2PN zygote	2.8 ± 2.9	3.8 ± 3.1	<0.01
Number of good blastocysts	0.5 ± 0.9	1.2 ± 1.6	<0.001
Rate of OPU = 0, %	2.6 (4/156)	2.6 (4/156)	1
Rate of MII 0, %	5.9 (9/152)	2.6 (4/152)	0.256
Rate of 2PN = 0, %	9.8 (14/143)	1.4 (2/148)	<0.01
ET rate/OPU cycles, %	41.0 (64/156)	62.8 (98/156)	<0.001
Rate of day 3 transfer, %	20.4% (24/98)	17.9% (28/156)	0.263
Number of embryos transferred/ET cycles	1.6 ± 0.90	1.7 ± 0.86	0.578
Implantation rate/OPU cycles, %	11.5 (18/156)	39.1 (61/156)	<0.0001
Implantation rate/embryo transferred, %	18.4% (18/98)	37.2% (61/164)	<0.01
Clinical pregnancy rate/OPU cycles, %	4.5 (7/156)	32.1 (50/156)	<0.0001
Live birth/OPU cycles, %	0.6 (1/156)	24.4 (38/156)	<0.0001

ET, embryo transfer; MII, metaphase II; OPU, oocyte pickup; PN, pronuclear.

Similar results were obtained for both groups B and C. In poor responders (Group B), although the number of MII oocytes (2.2 ± 1.8 versus 2.8 ± 1.9, *P* = 0.053) was not significantly increased, the number of oocytes retrieved (3.0 ± 2.2 versus 3.7 ± 2.2, *P* = 0.027), number of 2PN zygotes (1.7 ± 1.7 versus 2.3 ± 1.8, *P* = 0.015), and number of good blastocysts (0.4 ± 0.7 versus 0.9 ± 1.3, p=0.001) increased significantly in Cycle B. Subsequent ET rates (38.6 versus 59.0, *P* = 0.0127), implantation rates/OPU cycles (12.0 versus 31.3, *P <* 0.01), clinical pregnancy rates (2.4 versus 21.7, *P <* 0.001), and live birth rates (0 versus 18.1, *P <* 00001) increased significantly in Cycle B. Implantation rates/the number of embryos transferred (20.8% versus 34.7%) was higher in Cycle B; however, this was not statistically significant (*P* = 0.109). Similarly, the periods up to the trigger (12.9 ± 2.0 versus 21.3 ± 2.4, *P <* 0.001) and the gonadotropin dose (1687 ± 983 versus 2250 ± 974, *P <* 0.001) increased significantly in Cycle B. The mean leading follicle size (21.6 ± 4.1 versus 21.7 ± 2.5, *P* = 0.722) and E2 level (1093 ± 599 versus 1118 ± 557, *P* = 0.783) were not different between Cycles A and B, but the P4 level was significantly lower in Cycle B (0.62 ± 0.46 versus 0.38 ± 0.25, *P <* 0.001). The cancellation rates because of OPU=0 (4.8 versus 4.8, p=0.1) and MII=0 (8.9 versus 3.8, *P* = 0.328) were not different, but that because of 2PN=0 was significantly lower (13.9 versus 1.3, *P <* 0.001) in Cycle B (**Table 3**).

**Table 3:** Comparison of parameters and outcomes measured in poor responders (Group B).

	Cycle A	Cycle B	P
Period up to trigger, days	12.9 ± 2.0	21.3 ± 2.4	<0.001
Gonadotropin, IU	1687 ± 983	2250 ± 974	<0.001
Leading follicle, mm	21.6 ± 4.1	21.7 ± 2.5	0.722
Estradiol, pg/mL	1093 ± 599	1118 ± 557	0.783
Progesterone, ng/mL	0.62 ± 0.46	0.38 ± 0.25	<0.001
Number of oocytes	3.0 ± 2.2	3.7 ± 2.2	0.027
Rate of MII oocyte, %	73 ± 34	75 ± 28	0.617
Number of MII oocytes	2.2 ± 1.8	2.8 ± 1.9	0.053
Number of 2PN zygote	1.7 ± 1.7	2.3 ± 1.8	0.015
Number of good blastocysts	0.4 ± 0.7	0.9 ± 1.3	0.001
Rate of OPU = 0, %	4.8 (4/83)	4.8 (4/83)	1
Rate of MII 0, %	8.9 (7/79)	3.8 (3/79)	0.328
Rate of 2PN = 0, %	13.9 (10/72)	1.3 (1/76)	< 0.001
ET rate/OPU cycles, %	38.6 (32/83)	59.0 (49/83)	0.0127
Rate of day 3 transfer, %	22.9% (11/48)	21.3% (16/75)	0.827
Implantation rate/OPU cycles, %	12.0 (10/83)	31.3 (26/83)	< 0.01
Implantation rate/embryo transferred, %	20.8% (10/48)	34.7% (26/75)	0.109
Clinical pregnancy rate/OPU cycles, %	2.4 (2/83)	21.7 (18/83)	< 0.001
Live birth/OPU cycles, %	0 (0/83)	18.1 (15/83)	<0.0001

ET, embryo transfer; MII, metaphase II; OPU, oocyte pickup; PN, pronuclear.

In Group C, although the number of oocytes retrieved did not increase significantly between Cycles A and B (8.4 ± 6.2 versus 9.8 ± 7.7, *P* = 0.256), the number of MII oocytes (5.1 ± 3.8 versus 6.6 ± 4.0, *P* = 0.032), number of 2PN zygotes (4.0 ± 3.4 versus 5.4 ± 3.4, *P* = 0.015), and number of good blastocysts (0.7 ± 1.1 versus 1.6 ± 1.9, *P <* 0.001) increased significantly. Subsequent ET rates (39.7 versus 67.1, *P <* 0.01), implantation rates/OPU cycles (11.0 versus 47.9, *P <* 0.001), implantation rates/the number of embryos transferred (16.0% versus 39.3%, *P <* 0.005), clinical pregnancy rates (6.8 versus 43.8, *P <* 0.0001), and live birth rates (1.4 versus 31.5, *P <* 0.0001) increased significantly in Cycle B. The periods up to trigger (12.6 ± 1.5 versus 21.1 ± 2.2, *P <* 0.001) and gonadotropin dose (1614 ± 674 versus 1905 ± 548, *P* = 0.005) increased significantly in Cycle B. The mean leading follicle size (22.4 ± 4.2 versus 22.0 ± 3.1, *P* = 0.452) and E2 level (2281 ± 1446 versus 2220 ± 1462, *P* = 0.797) were not different between Cycles A and B, but P4 level was significantly lower in Cycle B (0.74 ± 0.73 versus 0.42 ± 0.24, *P <* 0.001). The cancellation rates because of OPU=0 (0 versus 0, p=1), MII=0 (2.7 versus 1.4, p=1), and 2PN=0 (5.6 versus 1.4, *P* = 0.209) were not different between the two cycles (**Table 4**).

**Table 4:** Comparison of parameters and outcomes measured in patients with poor-quality embryos (Group C).

	Cycle A	Cycle B	P
Period up to trigger, days	12.6 ± 1.5	21.1 ± 2.2	<0.001
Gonadotropin, IU	1614 ± 674	1905 ± 548	0.005
Leading follicle, mm	22.4 ± 4.2	22.0 ± 3.1	0.452
Estradiol, pg/mL	2281 ± 1446	2220 ± 1462	0.797
Progesterone, ng/mL	0.74 ± 0.73	0.42 ± 0.24	<0.001
Number of oocytes	8.4 ± 6.2	9.8 ± 7.7	0.256
Rate of MII oocyte, %	63 ± 25	74 ± 23	0.009
Number of MII oocytes	5.1 ± 3.8	6.6 ± 4.0	0.032
Number of 2PN zygote	4.0 ± 3.4	5.4 ± 3.4	0.015
Number of good blastocysts	0.7 ± 1.1	1.6 ± 1.9	<0.001
Rate of OPU=0, %	0 (0/73)	0 (0/73)	1
Rate of MII 0, %	2.7 (2/73)	1.4 (1/73)	1
Rate of 2PN=0, %	5.6 (4/71)	1.4 (1/72)	0.209
ET rate/OPU cycles, %	39.7 (29/73)	67.1 (49/73)	<0.01
Rate of day 3 transfer, %	26.0% (13/50)	13.5% (12/89)	0.106
Implantation rate/OPU cycles, %	11.0 (8/73)	47.9 (35/73)	<0.001
Implantation rate/embryo transferred, %	16.0% (8/50)	39.3% (35/89)	<0.005
Clinical pregnancy rate/OPU cycles, %	6.8 (5/73)	43.8 (32/73)	<0.0001
Live birth/OPU cycles, %	1.4 (1/73)	31.5 (23/73)	<0.0001

ET, embryo transfer; MII, metaphase II; OPU, oocyte pickup; PN, pronuclear.

## 4 Discussion

In this study, the protocol of delayed stimulation using the GnRH antagonist with progesterone and high-dose gonadotropin was better than other conventional COS protocols in terms of the number of MII oocytes, 2PN zygotes, morphologically good blastocysts, and the live birth rates in patients with recurrent ART failure. In poor responders, although the number of MII oocytes was not significantly increased, the number of oocytes retrieved, 2PN zygotes, and number of good blastocysts increased significantly. In patients with poor-quality embryos, although the number of oocytes retrieved did not increase significantly, the number of MII oocytes, 2PN zygotes, and good blastocysts increased significantly. These results indicate that this protocol increases the number of retrieved oocyte in poor responders, and MII oocytes in patients with poor-quality embryos. In addition, the result of increasing number of good blastocyst indicates that this protocol contribute to improve the quality of the oocytes. There are many reports describing the efficiency of a delayed-start GnRH antagonist protocol in patients with a low ovarian response (3, 7–10, 12, 13). In particular, the delayed-start GnRH antagonist with an estrogen priming protocol, first described by Cakmak et al. (7), seems effective based on a recent meta-analysis (13). However, the European Society of Human Reproduction and Embryology guidelines (21) considered the delayed-start GnRH protocol as a conditional low recommendation. Although the recommendation of the European Society of Human Reproduction and Embryology guidelines is low, the delayed-start GnRH protocol, compared with other conventional COS, may be effective for poor responders to increase retrieved oocytes and clinical pregnancy rate and decrease the cancellation rate (3, 4, 7–10, 12, 13), which is similar to the result in this study. Administration of GnRH antagonists in the early follicular phase is thought to suppress follicle-stimulating hormone levels, which allows growth of smaller antral follicles and halts the time for larger antral follicles (13, 22, 23). Suppression of early luteinizing hormone (LH) rise in patients with poor ovarian response may play a role in the improvement of outcomes (3). It leads to synchronized follicle growth and increased numbers of MII oocytes and good blastocysts, thereby increasing the chances of ET and live birth. Additionally, this protocol allows the use of more gonadotropin in terms of both dose and duration. Administration of GnRH antagonists in the early follicular phase suppresses the endogenous follicle-stimulating hormone and LH, which increases the requirement for gonadotropin.

A recent meta-analysis (13) has shown that a delayed-start GnRH antagonist protocol is more effective than long GnRH agonist, microdose GnRH agonist, multiple-dose GnRH agonist, GnRH antagonist, or GnRH antagonist/letrozole in patients with poor ovarian response. The main advantage is that GnRH antagonists have an early pituitary suppression and recovery from suppression compared with other protocols (4). However, the disadvantages of this protocol include a longer treatment time and a higher dose of gonadotropin. Frankfurter’s protocol is an original delayed GnRH antagonist protocol combined with progesterone. Progesterone-mediated LH surge suppression is currently used as a progestin-primed ovarian stimulation protocol. This suppression is mediated by an increase in dynorphin and GABAA receptor signaling acting through kisspeptin neurons in the anteroventral periventricular nucleus of the hypothalamus (23). Therefore, we hypothesized that GnRH antagonist administration might decrease progesterone supplementation. To overcome this disadvantage, earlier and lower doses of GnRH antagonists were administered (day 3 and one administration of 3 mg cetrorelix acetate) compared with Frankfurter’s protocol (days 5–8/9–12 and two administrations of 3 mg cetrorelix acetate) (3). As a result, the stimulation period was shorter (11 versus 12 days) and the gonadotropin dose was lower (2097 versus 5400 IU) than those in Frankfurter’s protocol. The number of retrieved oocytes (6.3 versus 4.5), number of zygotes (3.6 versus 2.5), and live birth (ongoing pregnancy) rate (21.8% versus 25.0%) were similar in both protocols. Based on these results, our protocol is as effective as Frankfurter’s protocol and addresses the disadvantage.

These findings were especially evident in poor responders but there have been few reports describing the efficiency in patients with poor-quality embryos. Younis et al. (4) investigated the efficiency of GnRH antagonist pretreatment among patients with two intact ovaries, age <39 years, body mass index 18–32 kg/m^2^, and a normal uterine cavity, excluding polycystic ovary syndrome, severe endometriosis, low ovarian reserve, thyroid disease, diabetes mellitus, significant hyperprolactinemia, and hypogonadotropic-hypogonadism. They concluded that pretreatment with a GnRH antagonist improved the meiotic status of retrieved oocytes and their competence for normal fertilization by suppressing serum follicle-stimulating hormone and LH levels while COS (not significantly). Our results in patients with poor-quality embryos (Group C) are similar to those in their report. Therefore, in addition to poor responders and other patients with ART failure, including those with poor-quality embryos, early follicular-phase GnRH antagonist administration is effective in increasing the number of mature oocytes, 2PN zygotes, and good blastocysts and the chance of live birth. The most significant difference in reasons for cancellation was 2PN=0. It is difficult to explain why 2PN increased in Cycle B only because of the increased oocyte counts. This indicates that this protocol may have a positive effect on the quality of oocytes, as previously described by Younis et al. (4). Further research, including basic research, is necessary to confirm this hypothesis.

The limitations of this study are its retrospective design, the various protocols in Cycle A, and the subsequent advantage of Cycle B. It was previously reported that a change in COS protocol for the second cycle may affect the outcome in both positive and negative ways regarding oocyte recovery and the total number of mature oocytes/embryos (7, 24, 25). In this study, the median number of previous OPU cycles was three, so personalized intervention, such as the timing of OPU after the trigger, can improve the MII rate and decrease cancellation. In fact, the size of the leading follicle on the trigger day and the cancellation rate because of OPU=0 and MII=0 were not different between Cycles A and B. In addition, an interval of more than 6 months between the two cycles was excluded to reduce the chance of improved outcomes in the latter cycle as a result of the intervention using new technology. Although the data were not shown, Frankfurter et al. (3) reported that the type of initial COS protocol used did not affect the outcomes. The limitations of this study are not completely eliminated, and the facts mentioned above support its reliability. In the future, a randomized prospective trial is desirable to evaluate the effectiveness of this protocol. To further evaluate the effectiveness of the GnRH antagonist combined with MPA, a comparison of the delayed GnRH antagonist protocol with or without MPA in the early follicular phase is desirable.

In conclusion, the original delayed-start GnRH antagonist with progesterone and high-dose gonadotropin protocol is effective and useful as an alternative protocol in patients with recurrent ART failure, both in poor responders according to the Bologna criteria and patients with poor outcomes because of poor-quality embryos. Although it has disadvantages compared with other COS protocols, such as a longer treatment period and a larger dose of gonadotropin, it has an advantage in terms of the number of oocytes retrieved, number of morphologically good blastocysts, and the live birth rate. However, further research is required to confirm this finding.

## Data availability statement

The raw data supporting the conclusions of this article will be made available by the authors, without undue reservation.

## Ethics statement

The studies involving humans were approved by Institutional Review Board of Takeuchi Ladies Clinic/Center for Reproductive Medicine. The studies were conducted in accordance with the local legislation and institutional requirements. Written informed consent for participation was not required from the participants or the participants’ legal guardians/next of kin because this study is a retrospective study which only hundle anonymized data. So that we did not take individual written consent. But we announced this study in displays and on the hospital’s homepage and provided an opt-out option for patients. No patient offered opt-out.

## Author contributions

KT: Conceptualization, Data curation, Project administration, Writing – original draft, Investigation, Methodology. YO: Conceptualization, Data curation, Project administration, Writing – original draft, Formal Analysis. TI: Conceptualization, Data curation, Writing – review & editing. YKuw: Resources, Writing – review & editing. YKur: Resources, Writing – review & editing. YF: Resources, Writing – review & editing. YM: Resources, Writing – review & editing. MT: Resources, Writing – review & editing. HM: Resources, Writing – review & editing.
